# Development of broad spectrum mycosporine loaded sunscreen formulation from *Ulva fasciata* delile

**DOI:** 10.1051/bmdcn/2019090317

**Published:** 2019-08-27

**Authors:** Saurabh Bhatia, Satish Sardana, Ajay Sharma, Celia Bertha Vargas De La Cruz, Bhupal Chaugule, Laleh Khodaie

**Affiliations:** 1 Amity institute of pharmacy, Amity University Haryana Gurgaon Haryana-122413 India; 2 Amity Institute of Pharmacy, Amity University Madhya Pradesh Maharajpura Gwalior (MP)-474005 India; 3 Departemnt of Pharmacy and Biochemistry, Centro Latinoamericano de Enseñanza e Investigación en Bacteriologia Alimentaria, National San Marcos University Lima Peru-15081; 4 Poona college of Pharmacy, Bharati Vidyapeeth Deemed University Pune Maharashtra-411045 India; 5 Faculty of Persian Traditional Medicine, Tabriz University of Medical, Sciences Tabriz-51368 Iran

**Keywords:** Ulva, Gel, Sunscreen, Ultraviolet, UV-B, Mycosporine amino acid, Broad spectrum, *Ulva fasciata*

## Abstract

Ba.ckground: Sunscreen formulations primarily offer protection against UV induced damages however nowadays it also maintains skin natural physiological conditions. Current global market is flooded with numerous sunscreen products which offer protection to skin against several UV induced damages. However most of these sunscreen formulations offers narrow spectrum protection against UV and also suffer from stability as well as toxicity related issues.

Methods: Present work aims to isolate mycosporine amino acid (Mgy) from green alga namely *Ulva fasciata (U. fasciata)* and study its sunscreen potential against widely used domestic marketed formulation. Stability evaluations were also performed for almost 90 days.

Results: Results demonstrated that the isolated compound, mycosporine glycine (Mgy) preserved physicochemical properties of the product and offered good stability for all formulations throughout the experimental period. Furthermore, Mgy loaded carbopol gel showed better sunscreen protection against marketed formulation in a concentration dependent manner. (7.709).

Conclusion: (6.806) Novel Myg loaded gel was proved to demonstrate several quality characteristics that may unlock new prospects for the production of more efficient, safe, and economic skin-care products.

## 1. Introduction

Ultraviolet (UV) irradiation and its interaction with living system is a critical issue from several years. UV irradiation represents UV region (280 to 400 nm) which is divided in to three components: UV-B irradiation (280 to 315 nm), UV-A (315-400 nm) and UV-C (200 to 280 nm) [1–5]. UV-C is not of biological relevance as it’s totally absorbed by the atmosphere. Due to global warming (ozone depletion) there is a marked increase in the UV-B (7-8 W mK^2^) irradiation. Marked increase in UV-A (45-50 W mK^2^) also observed in some of the regions [6–9]. This is considered as one of the major global environmental threat which can increase injurious effects of UV radiation over health. Thus utilization of sunscreen agents is always encouraged globally to prevent chances of skin injuries caused by UV radiations. Most of the marketed formulations are specifically prepared to block UV-B absorption. These formulations not only prevent the absorption but also reflect UV-B. One of the most common approaches to evaluate potential of the sunscreen is Sun Protection Factor (SPF). SPF of any formulation can be calculated by evaluating its potential in inhibiting erythema (redness due to inflammation) and edema (swelling or inflammation). These skin reactions can be noticed 24 h after exposing skin against UV radiations [10–17]. UVA (315-400 nm) is having longer wavelength, thus it’s not efficient in inducing erythema or any other skin reactions whereas UVB (280-315 nm) is required to induce erythema within less period of time. Present sunscreen formulations which are already available in the market offer protection mainly against UVB and only few formulations are broad-spectrum UVA filters. Additionally these sunprotective compounds are more sensitive against other excipients present in the formulations which causes several stability problems. So the current Indian market requires the most compatible, stable and broad spectrum compounds or formulations which can provide a protective screening shield to the human skin.


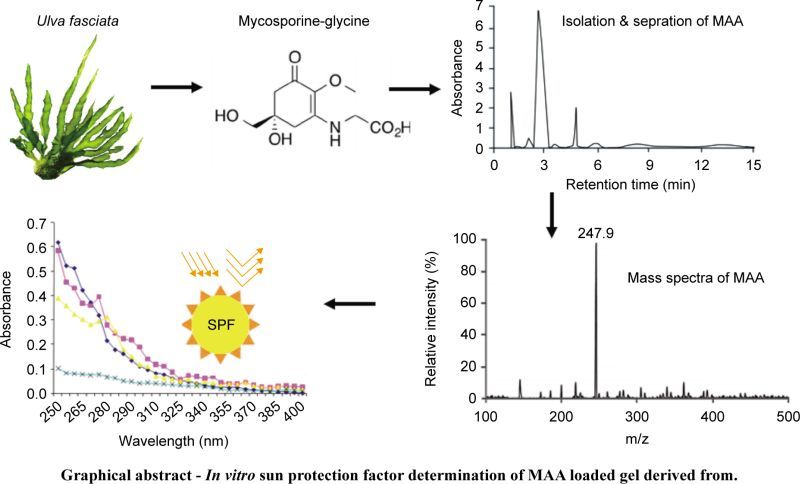


**Graphical abstract – *In vitro* sun protection factor determination of MAA loaded gel derived from.**

Marine organisms are the rich source of bioactive compounds which are having extensive range of biological activities such as antibacterial, anti-inflammatory, antifungal, antioxidant, antiulcer, sunprotective, immunomodulatory and anti-microbial algal compounds [18–30]. Algal resources or seaweed are considered as rich source of therapeutic compounds which are having potential and diverse biological activities.

These compounds once identified, isolated and toxicological verified against designed pharmacological models can be considered as good candidate for formulation and development. Recent reports suggested the presence of unique class of sun protective compounds present in algae called as mycosoorine amino acids (MAAs). These MAAs are comparatively more present in seaweeds which can be considered as a vital source for sunprotective compounds. *Ulva fasciata,* a green marine alga belonging to family Ulvaceae, is widely available in east and west coast of India. The first report of Ulva *(U. lactuca* L.) was from the India which was evidenced by Boergesen (1934) [31]. This alga is commonly found in the intertidal region along the coastline typically lithophytic or epiphytic. So far 14 species have been evidenced from Indian origin [32]. *Ulva* is not reported so far for its sun protective action but still carries photo-adaptive mechanisms to protect against intense sun radiations. This type of mechanism includes radical scavenging potential and triggering the synthesis of sun protective compounds (secondary metabolites) such as mycosporine-like amino acids (MAAs) [33–39]. In our previous study we have reviewed and investigated the major characteristics of these water-soluble compounds namely MAAs (309 to 360 nm) and explore *in-vitro* sun protection of Porphyra-334 from *Porphyra vietnamensis.* Ulva contains high amount of carbohydrate, protein, lipid, moisture, dietary fiber, vitamin C and ash content. Moreover this green alga is also enriched with minerals and amino acids. A unique class of amino acid mainly mycosporine glycine (Mgy) which is rarely available in green alga has been identified in this study. Current study is to identify, isolate and evaluate stability of sun protective compounds derived from *U. fasciata.* Furthermore isolated compound was further assessed for its sun screening potential against “Lotus gel” [40, 41].

## 2. Materials and methods

### 2.1. Materials required

Lotus Herbals Safe Sun UV Screen Matte Gel, SPF 50, was considered, acetic acid (Merck), triethanolamine (Merck), methanol (Merck), ethanol (Merck), *U. fasciata* was collected from Harihareshwar, Maharashtra, India (Latitude and longitude coordinates are: 17.994234, 73.025803). Alga was identified and authenticated by Prof. B. B. Chougule (Taxonomist), Department of Pharmacognosy, Poona College of Pharmacy, Bharati Vidya Peeth University, Pune, Maharashtra-411038, India. A voucher specimen has been deposited in the herbarium at the Department of Botany, Savitribai Phule Pune University, Pune, Maharashtra, India under the voucher specimen number UH1.

The sufficient amount of fresh Ulva fasciata sample (100 g) was preserved by glutaraldehyde (3%), and sample was entirely washed before its usage. Ethanolic fraction was isolated from dried algal material which was kept at 55 °C in an air dryer for almost 48 h. Usually it takes longer time in drying of algal material then terrestrial plant material drying.

**Fig. 1:**
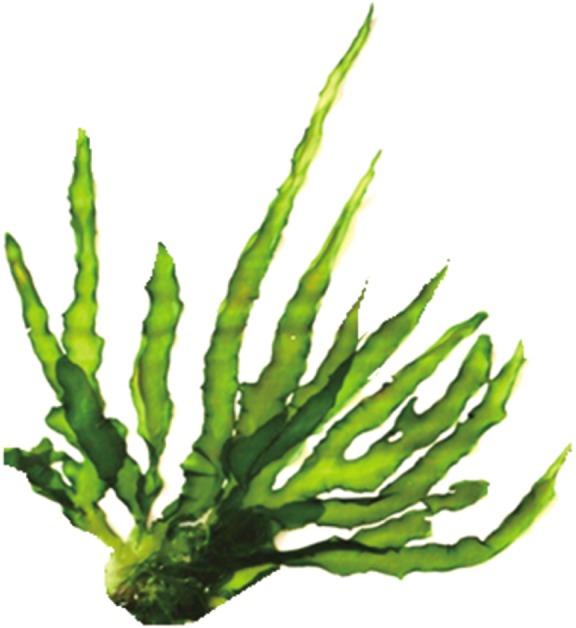
Ulva fasciata (sample was collected from Harihareshwar, Maharashtra, India; global location coordinates *i.e.* latitude and longitude coordinates are: 17.994234, 73.025803); scale bars: 2 cm.

Wiley mill (Model 4276-M, Thomas Scientific, USA) was used to powder dried algal material. Powdered algal material was passed from 20 mesh sieve. Algal material was stored in transparent plastic bags and sealed properly. Dried algal material was utilized for extraction purpose. Several morphological characteristics of seaweed have been evaluated such as plant height (47 cm), plant width (12-13 cm), width of each lobe (2.5-4.3 cm), shape (Linear), attachment (circular disc), stipe (absent), margin (undulate), thickness of thallus in middle area (68-107 μm), thickness of thallus in basal area (69-172 μm) distance between two layers middle region (3.1-4.3 μm), length of rhizoid (31.2-65.5 μm), Dimensions of vegetative cells across the surface (13-17 μm), microscopic teeth (absent) (Fig. 1). Alga was identified by using Krishnamurthy & Joshi. (1969) [47]

### 2.2. Extraction and isolation procedure

Twenty gram of dried algal material was powdered and treated with a mixture of ethanol and water (75: 25, v/v) followed by treatment with chloroform. Non polar fraction was removed whereas polar fraction was lyophilized ultimately to yield two gram of a pale yellow powder. Weighed quantity of powder was re-treated with 80% ethanol.

### 2.3. UV-Vis analysis

UV absorbance of aliquot was determined by using UV-Vis spectrophotometer (UV-spectrophotometer: Jasco V 530, Jasco).

### 2.4. Isolation of bioactive compound

To isolate bioactive compound, powder derived from the initial procedure was eluted through a silica gel column (60-120 mesh) loaded with 20% methanol +0.2% acetic acid. TLC analysis was done to ensure the homogeneity of loaded fractions. Out of 2 gram of loaded sample 377 mg of pale yellow powder was obtained. Percentage yield [(Wt of bioactive compound: 377 mg) / Wt of raw material: 2000 mg) ×100] was calculated. Resulting fraction was evaporated and lyophilized till dryness to ultimately yield 270 mg of a colorless powder.

### 2.5. HPLC analysis

To ensure purity of the sample isolated fraction was further analyzed by HPLC with PDA detector (SPD-M30A, 85 mm optical path length, 0.4 × 10^−5^ AU noise level) with ODS (RP-18 column) and a guard (ODS, 5 μm, 4.0 × 10 mm). 20% methanol +0.2% acetic acid (mobile phase) was run at the rate of one millilitre per minute. By using auto-injector fifty micro litre sample was injected into the HPLC column. 20 % methanol +0.2% acetic acid was loaded, which showed retention time of 2.8 min. To record absorption spectra of sample, detector was scanned from 200 to 450 nm. HPLC peak was recorded at wavelength of MAA was 320 nm. Pure sample (MAA) was collected by using fraction collector which was again lyophilized. The whole investigation was performed on highly polar water soluble compound (mycosporine glycine) that absorbs strongly in the 10 nm to 400 nm (UVA region) [42].

### 2.6. Mass analysis

MAA was further analyzed by using liquid-mass spectrometry. (LC-MS) (Shimadzu LC-MS-2020) [43]

### 2.7. Development of MAA loaded carbopol gel

MAA loaded Gel was prepared by using 1% of carbopol (carbopol 934), 1% of MAA, EDTA (0.03%), propylene glycol (3%) as major ingredients. Whole mixture was dissolved in water and heated upto 5 min at 50 °C. Prepared mixture was kept for 48 h in dark place. To achieve the uniform consistency of gel and to stabilize its pH, a crosslinker (triethanolamine) was added in a drop wise fashion.

### 2.8. Physiochemical properties of MAA loaded gel

Morphological characterization of gel: Physical appearance and color was monitored by visual observations over period of time from 1-80 days.

pH: pH of drug loaded gel was determined by means of pH meter (calibrated). This was done at various at different twenty days time interval (1, 20, 40, 60, 80 days) to further check any pH variation by the time.

Viscosity: Brookfield viscometer (Synchrolectric Viscometer, Stoughton, MASS 02072, USA, at 50 rpm, spindle number 5) was used for measuring apparent viscosities of MAA loaded carbopol gel at 55 °C.

Gelling strength (GS), gelling temperature (GT), melting temperature (MT), and molecular weight (MW) determination, spread ability (SA): GS of the MAA loaded carbopol gel was evaluated at 10 °C by means of model TA-XT2 Texture analyzer (Stable Micro System, Surrey, UK). Other parameters such as GT, MT, MW were determined by methods introduced by Craigie and Leigh [44] and Rochas and Lahaye [45]. SA was determined by measuring spread diameter of one gram of gel. This was performed by keeping one gram of gel between two plates of dimensions 20 cm each.

Skin erythema and edema evaluation: Male Swiss mice of weight range 25 g was used for this study. Optimal conditions were maintained at a controlled temperature (22 ± 2 °C) for 12-hours light/dark cycle. To prevent food interference with reference/test drugs absorption, eight hours before each experiment animals received only water. The experimental studies were conducted with the consent of the procedure by the local Institutional Ethics Committee-IAEC/ABMRCP/2016-2017/11. Prepared gel and gel base as control were applied after removing hairs of the selected animal. Visual observations were made after an interval of seven days of gel application [46].

### 2.9. *In vitro* sun protection determination method

Selected formulation was evaluated for sun protective efficiency by utilizing simple and rapid *in vitro* sun protection determination method [25, 26]. Marketed Lotus gel formulation was selected as reference formulation. Three MAA loaded carbapol gel with different dilutions from 50-150 μ/ml were prepared in methanol. UV spectrophotometer was used to measure absorbance of all dilutions [46]. This was recorded at 5 nm intervals from 250-400 nm. Each determination was made in triplicate (Mean ± sd). The observed absorbance values were calculated according to the equation given below.

$$\mathrm{SPE}\;=\;\mathrm{CF}\;\times \;\sum_{100}^{250}\mathrm{EE}\;(\lambda )\;\times \;[(\lambda )\;\times \;\mathrm{Abs}\;(\lambda )$$

Where CF = 10 (Correction factor), EE (λ) = Erythemogenic effect of radiation with wavelength λ, Abs. (λ) = Spectrophoto-metric absorbance values of a solution of the preparation at wavelength [10, 13, 46].

The aliquots prepared were scanned between 290 and 320 nm, and the obtained absorbance values were multiplied with the respective EE (l) values. Then, their summation was taken and multiplied with the correction factor (10) [10].

## 3. Results and discussion

### 3.1. Characterization of isolated MAA

**Fig. 2:**
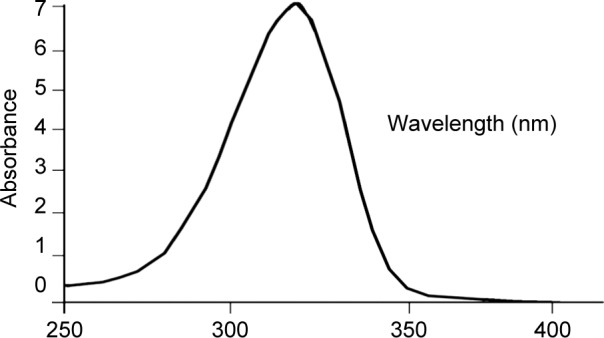
Detection of MAA (320 nm) by UV from the ethanolic fraction of Ulva.

From taxonomic features (Fig. 1) it was confirmed that harvested alga was *U. fasciata.* Average percentage yield of MAA is 13.5%. Isolated bioactive compound was characterized initially by UV spectrophotometer and HPLC-PDA detector. UV analysis revealed appearance of sharp peak with an extinction coefficient of 31,177 M^−1^ cm^−1^ which was obtained at 320 nm (Fig. 2). From UV/Vis, LC-MS and HPLC analysis it was almost clear that isolated compound was mycosporine-glycine (Mgy) with λmax 320 nm, retention time (RT) 2.8 and mass charge ratio (m/z) 247.9 as depicted in Fig. 2. Because of the unavailability of standard marker compounds and challenge involved in the synthesis of MAA, most of the MAA are identified by their RT, M/Z and λmax. On the other side synthesis of MAA is expensive thus characterization of MAAs by these techniques could be an important contribution in the field of development of an active sunscreen compound.

**Fig. 3:**
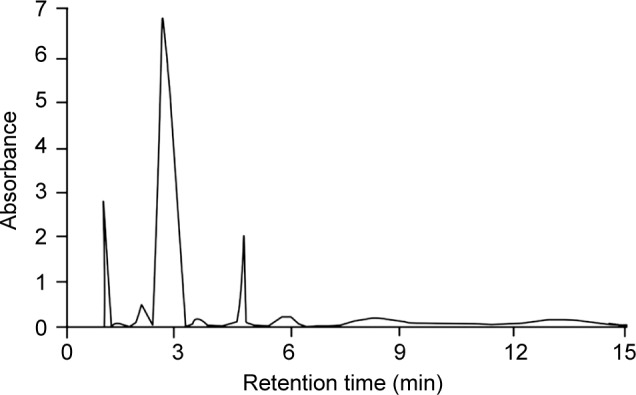
HPLC- PDA study of partially purified mycosporine glycine (Mgy) derived from Ulva sp. presenting the prominent peak at RT 2.78 min with λmax 320 nm.

**Fig. 4:**
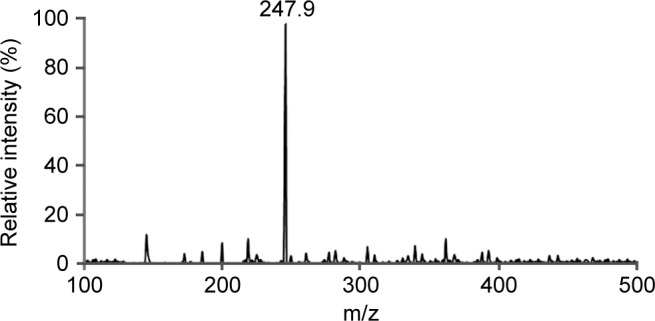
Mass spectra purified compound (mycosporineglycine), showing a typical peak at m/z 247.9.

HPLC- PDA study of the fraction of bioactive compound (MAA) has shown a prominent peak at 2.8 min (Fig. 3) with a UV absorption maximum (UVλmax) at 320 nm (Fig. 2). LCMS analysis of HPLC-PDA purified MAA showed a sharp ion peak of a protonated molecule ([M + H]+) at m/z 247.9 (Fig. 4).

### 3.2. Physiochemical properties of MAA loaded gel

Physiochemical properties of formulated gel in comparison with marketed formulation (Lotus gel) are mentioned in Table 1. It was observed that all the formulations showed characteristics such as appearance (white color) and homogeneity. As far as other parameters are concerned GSg (377 ± 2.4), GT (27.8 ± 0.1), MT (71.7 ± 0.2), cp (62.7 ± 0.3), MMS (21.127 ± 1.1), pH (6.2 ± 0.07) and SD (57 ± 0.1). There was no change in GSg, GT, MT, cp, MMS, pH and SD over a period of time interval 0, 30, 60, 90 days. Spread ability value suggested that gels can be easily separated by application of little shear. After one minute of time prepared gel showed good results. Skin erythema and edema studies indicated that prepared formulations didn’t caused any sign of dermatological reactions.

**Table 1:** Comparative Evaluation of MAA-Carbopol gel against Marketed formulation.

Parameters	MAA-Carbopol gel				*Lotus* gel (Marketed Preparation)
	0 day	30 days	60 days	90 days	
GSg (g/cm^2^)	377 ± 2.4	361 ± 1.3	377 ± 2.4	371 ± 2.1	417 ± 2.1

GT (°C)	27.3 ± 0.2	26.1 ± 0.1	26.8 ± 0.3	27.1 ± 0.1	25.4 ± 0.2

MT (°C)	71.7 ± 1.0	70.1 ± 1.3	71.3 ± 1.2	71.6 ± 1.1	67.1 ± 0.1

Apparent Viscosity (cp) at 80 °C	62.7 ± 1.3	61.7 ± 1.2	61.7 ± 1.5	61.8 ± 1.2	58.7 ± 0.2

MMS	21.1 ± 1.1	20.1 ± 1.0	22.1 ± 0.2	21.1 ± 1.1	17.1 ± 1.5

Ph	6.2 ± 0.07	6.2 ± 0.02	6.1 ± 0.04	6.1 ± 0.03	6.1 ± 0.01

Appearance	White	White	White	White	White

Homogeneity	Good	Good	Good	Good	Green

Spreading diameter (SD) after one minute (mm)	57 ± 0.1	58 ± 0.1	57 ± 0.2	58 ± 0.1	62 ± 0.2

Gelling strength (GS), gelling temperature (GT), melting temperature (MT), molecular weight (MW) determination, spread ability (SA), mycosprine amino acid (MAA), MMs (Molecular mass), Viscosity(cp), Spreading diameter (SD).

### 3.3. *In vitro* sunscreen potential of MAA loaded gel

**Fig. 5:**
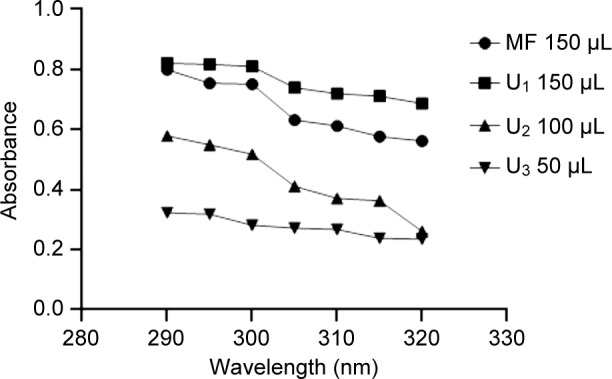
UV absorbance values of marketed Lotus gel and Mgy loded carbopol gel at different concentrations of MAA; U1-U3 (different conc. of MAA).

In present work a green alga harvested from Indian coast namely *Ulva.* Ethanolic extract *U. fasciata* was considered as a source for sun protective compound which was assessed for their UV absorbance potential against marketed formulation namely *Lotus* gel. Based on the results, it was concluded that UV screening potential of MAA loaded formulations was increased in a concentration dependent manner (50-150 μl) as mentioned in Table 2. Absorbance values of bioactive compound and marketed formulation at different concentrations (50-150 μl) were considered to calculate the *in-vitro* sun protection factor. This calculation was done by using the erythemogenic effect values (EE values). These values are relevant only for the UV-B (290-320 nm). As represented in Fig. 5 and table 2 MAA loaded Ulva gel was more active in screening UV radiations in comparison to standard Lotus gel. It was proven that MAA loaded Ulva gel broadly prevents UV radiations, signifying more potential against *Lotus* gel.

**Table 2:** SPF values using different concentrations of marketed Lotus gel formulation and Mgy loaded carbopol gel. (n = 3, mean ± sd)

Sr. No.	EE effect	Wavelength	Marketed formulations				MGY loaded test formulations			
			Lotus (150 μL)		U_1_ (150 μL)		U_2_ (100 μL)		U3 (50 μL)	
	EE	Abs.	Abs.	SPF	Abs.	SPF	Abs.	SPF	Abs.	SPF
1	0.021	290	0.799		0.821		0.579		0.323	
2	0.127	295	0.754		0.817		0.549		0.318	
3	0.266	300	0.751		0.810		0.518		0.281	
4	0.327	305	0.632	6.806	0.739	7.709	0.411	4.502	0.272	2.802
5	0.181	310	0.612		0.720		0.371		0.267	
6	0.057	315	0.577		0.711		0.363		0.238	
7	0.032	320	0.562		0.687		0.261		0.235	

SPF: sun protection factor, EE: erythemogenic effect AM: marketed lotus (150 μ), *Ulva* (U_1_-U_3_) freshly prepared gel from concentration (50-150 μl/ml).

## 4. Conclusion

Several reports have confirmed the presence of MAA among different marine sources and their fundamental way to protect marine organism against UV induced stress. Marine organisms are more vulnerable against UV induced injuries because seawater absorbs light much more strongly than air does and effects of ultraviolet radiation are greater in the coastal zone than in inland areas, due to reflection of sunlight from sand and the sea surface. Thus these marine organisms have various photo-adaptive mechanisms to protect against UV induced stress conditions. MAA is a family of amino acids which are diversely present among different marine organism especially seaweeds to protect them against photo-oxidative stress induced by UV irradiations. Thus in present work we have successfully isolated and formulated Mgy to test its sunscreen potential against marketed formulation. Results obtained from this work permit the conclusion that this sun-protective formulation constitutes the most prominent and potent amino acid which can in future become a promising molecule in UV-related dermatological ailments.

## Conflicts of interest statement

The authors wish to disclose no conflicts of interest.
